# Development and Validation of the Teen Dating Aggression Measure Among Canadian Youth

**DOI:** 10.3389/fpsyg.2021.763210

**Published:** 2021-12-14

**Authors:** Ryan J. Persram, Tracy K. Y. Wong, Luis Francisco Vargas-Madriz, Chiaki Konishi, Nicole S. J. Dryburgh, Melanie A. Dirks, Alexa Martin-Storey, Wendy Craig

**Affiliations:** ^1^Department of Educational and Counselling Psychology, McGill University, Montréal, QC, Canada; ^2^Department of Psychology, McGill University, Montréal, QC, Canada; ^3^Department of Psychoeducation, Université de Sherbrooke, Sherbrooke, QC, Canada; ^4^Department of Psychology, Queen’s University, Kingston, ON, Canada

**Keywords:** dating violence, adolescents, aggression, teen dating violence, adolescent dating aggression

## Abstract

Teen dating violence (TDV) victimization is a traumatic experience that can have adverse consequences for adolescents. Current measures that assess TDV do not fully distinguish between psychological and relational forms of aggression, nor do they capture aggressive acts that are common within adolescent relationships. The purpose of this study was to evaluate the psychometric properties of the Teen Dating Aggression Measure (TeDAM) using a sample of 730 Canadian adolescents (*M* = 15.88 years, *SD* = 1.23). The measure is an expansion of the Conflict in Adolescent Dating Relationships Inventory and includes items that describe other forms of violence such as coercion and control, along with more traditional indicators of dating violence (e.g., sexual aggression). Factor analyses yielded three factors, namely psychological aggression, sexual and physical aggression, and relational aggression, which were correlated with more frequent cannabis and alcohol use as well as rape myth acceptance. These results provide initial support for the utility of the TeDAM for assessing TDV with adolescents.

## Introduction

Adolescence represents a developmental period marked by increased interest and involvement in extrafamilial romantic relationships with same or other-gender partners (Collins et al., 2009; Furman and Collins, 2009). Although teen dating is typically characterized by affectionate and anticipated or actual sexual behaviors, violence, and aggression can also occur (Wolfe et al., 2001; Collins et al., 2009). This type of aggression and violence is commonly referred to as teen dating violence (TDV) and is a prevalent social problem. In a recent study using the Health-Behavior in School-Aged Children (HBSC) survey, Exner-Cortens et al. (2021) found that one in three Canadian teenagers had some experience with dating violence in the past year. In particular, adolescents reported experiencing psychological aggression the most (27.8%), followed by cyber aggression (17.5%) and physical aggression (11.8%) (Exner-Cortens et al., 2021). Moreover, national statistics indicate that 20% of police-reported incidences included some form of dating violence among 15–24-year-olds (Statistics Canada, 2018). Finally, youth victims of TDV are more likely to experience adverse outcomes such as increased depression and anxiety (Garthe et al., 2021), engage in more substance abuse and risky sexual behavior (Alleyne et al., 2011), and are at greater risk for future intimate partner violence (IPV) (Exner-Cortens et al., 2013). Despite being a public health concern, current measures of TDV are limited for various conceptual and methodological reasons. To address these limitations, the purpose of this study was to evaluate the psychometric properties of a more expansive measure that evaluates TDV victimization more broadly.

Teen dating violence is conceptually similar to IPV observed among adult populations (Espelage et al., 2020). For example, TDV incorporates actions and behaviors that are intended to hurt or manipulate a partner’s social relationships and can be manifested in different ways (Linder et al., 2002; Breiding et al., 2015; Centers for Disease Control and Prevention, 2020). Instruments that assess TDV typically measure three types: (a) physical, (b) sexual, and (c) psychological violence (Smith et al., 2015; Exner-Cortens et al., 2016a,b). Physical dating violence refers to a range of behaviors in which an individual experiences physical injury such as hitting and kicking (Breiding et al., 2015). Sexual dating violence refers to behaviors in which a partner forces or attempts to force the partner to engage in physical or non-physical sexual acts (Breiding et al., 2015). Psychological dating violence denotes behaviors meant to humiliate or control the partner (e.g., name calling, restricting access to friends) (Breiding et al., 2015). However, what is unique to TDV is that it can differ based on the types of aggressive behaviors and actions that often take place amongst adolescent peer groups that may not be particularly salient among adults.

In general, victimization resulting from TDV is linked to various negative outcomes. For example, experiencing physical or verbal TDV is associated with recent alcohol and marijuana use (e.g., Parker et al., 2016). Moreover, adolescents who have been exposed to sexual assault have differential perceptions of rape myth acceptance, which refers to a stigmatic set of beliefs that victims are at fault for their assault or exposure to dating violence (e.g., Edwards et al., 2011; Dworkin et al., 2017). Subscales of rape myth acceptance include rape denial, which refers to attitudes reflective of victim-blaming or not believing rape victims, and traditional gender expectations, which represent typical roles of men and women in relationships (Dworkin et al., 2017). In Dworkin et al. (2017), positive associations between the two rape myth acceptance subscales and each of depressed mood and alcohol use among a sample of adolescents were evident. More broadly, positive associations were observed between rape myth acceptance as an overall measure and sexual dating aggression (Reyes and Foshee, 2013). Thus, the consequences of being victimized by a romantic partner has an effect on the ways in which adolescents might cope with and perceive such experiences.

Currently, there are several measures that assess TDV, each of which evaluate different aspects of teen dating perpetration and victimization (see Exner-Cortens et al., 2016a,b). A comprehensive review by Exner-Cortens et al. (2016a,b) highlights dating violence behaviors and attitudes as distinct themes for measuring TDV. Despite the breadth of these measures, there are two main limitations to note among them. First, many of the measures are adapted from adult scales that do not fully capture the adolescent experience (e.g., Conflict Tactics Scale; see Straus, 1979; Cascardi et al., 1999). Although adult-based dating violence measures could be adapted for use with adolescents, the nature of adapting remains problematic. From a developmental perspective, adolescent and adult intimate relationships differ in important ways (Knox et al., 2009). For example, adolescents are less likely to live with and be economically bound to their partner (Knox et al., 2009). Moreover, adolescents are also more likely to disclose victimization experiences with peers than adults, who are more likely to seek help from professionals (Knox et al., 2009). Furthermore, adolescents are likely to remain in contact with the partner because they often go to the same school or even attend the same class (Knox et al., 2009). Due to these differences, adult-based dating violence measures might not adequately capture the uniqueness of TDV. For example, including items that capture economic threats (e.g., removal of economic support) or failing to assess relational aggression (e.g., spreading rumors to friends) would not adequately capture adolescent developmental. As such, we argue that they do not fully capture the everyday aggressive acts that might be more common to the adolescent dating experience. This is especially the case for relationally and psychologically aggressive acts, as they are common during adolescence and occur in both dating and peer relationships (e.g., Linder et al., 2002; Ellis et al., 2009).

In adolescence, relationally and psychologically aggressive acts are common among teen dating partners relative to physical aggression (e.g., Morelli et al., 2018; Dosil et al., 2020; Asghari et al., 2021). In addition, psychological aggression appears to occur more frequently than relational aggression in the context of adolescent romantic relationships relative to those between adults (Morelli et al., 2018; Dosil et al., 2020). Similar to the aforementioned TDV experiences, adolescent victimization based on psychological or relational acts are associated with greater psychological distress in romantic relationships (Jouriles et al., 2009; Goncy et al., 2017), increased emotional and behavioral problems (e.g., Leadbeater et al., 2008), and alcohol use (Schad et al., 2008). As such, the inclusion of aggressive acts that adolescents commonly see in their romantic partners beyond what has been developed for adults is necessary.

Second, adolescent-based measures of TDV tend to have poor psychometric properties. For example, the Conflict in Adolescent Dating Relationships Inventory (CADRI) is a widely used instrument to measure teen dating perpetration and victimization and has been validated in many countries, including Canada, the United States, Spain, Mexico, among others (Wolfe et al., 2001; Smith et al., 2015; Exner-Cortens et al., 2016a,b). In terms of its factor structure, the CADRI includes five first-order factors, including threatening behavior, physical abuse, relational aggression, verbal emotional abuse, and sexual abuse, each of load onto a second-order latent factor called abuse. Moreover, internal consistency and test-retest reliability were adequate across adolescent sex and grade (>0.70) for verbal/emotional and physical abuse, but weaker for threatening behaviors (0.54–0.73), relational aggression (0.16–0.69), and sexual abuse (0.36–0.59) (Wolfe et al., 2001). Subsequent validation studies offered only partial psychometric evidence for the CADRI. For example, Fernández-Fuertes et al. (2006) assessed the five-factor model for both TDV perpetration and victimization with Spanish adolescents using exploratory factor analysis (EFA) instead of confirmatory factor analysis (CFA). Hokoda et al. (2006) only considered the reliability of CADRI for both TDV perpetration and victimization with Mexican youth. Furthermore, although the short form of CADRI (CADRI-S) has demonstrated validity and reliability among general and high-risk adolescents, it has lower sensitivity compared with the full CADRI (Fernández-González et al., 2012). Importantly, the CADRI-S was assessed only from the perpetuator’s perspective (Fernández-González et al., 2012). Thus, the extent to which CADRI or CADRI-S adequately measures TDV victimization experiences remains unclear.

The Measure of Adolescent Relationship Harassment and Abuse (MARSHA) is a recent and comprehensive instrument that aims to evaluate different aspects of TDV perpetration (i.e., social control, physical abuse, sexual abuse, isolation, cyber control, intimidation) and victimization (i.e., privacy control, social control, physical abuse, sexual abuse, and intimidation (Rothman et al., 2020, 2021). This measure was initially developed from focus groups and interviews with a group of adolescents and young adults (11–20 years old, *M*_age_ = 16.4) (Rothman et al., 2020), and its reliability and validity were evaluated with a culturally diverse sample (11–20 years old) that has a mean age of 18 (Rothman et al., 2021). Given that the validation group consisted mainly of older adolescents and young adults, the extent to which MARSHA adequately captures TDV victimization experiences during the period of *adolescence* is inconclusive.

Previous reviews have addressed several gaps in extant TDV measurements (Smith et al., 2015; Exner-Cortens, 2018). For example, items that measure sexual violence are often neglected or limited, possibly because of pressure from schools to avoid sexual related topics (Smith et al., 2015; Exner-Cortens, 2018). Moreover, psychological aggression (e.g., insulting) is often measured in a way that parallels physical aggression, which limits understanding on the nuances that distinguish the two constructs (Follingstad, 2007). To address these gaps, this study validated a comprehensive TDV victimization measure that was extended from the CADRI measure. This measure considers not only fundamental TDV constructs (i.e., physical abuse, psychological abuse, and sexual abuse), but also those that have not been adequately evaluated, such as relational forms of violence and aggression. The validation of the new measure was conducted in Canada, where TDV remains an alarming significant public health problem among adolescents (Shaffer et al., 2021).

The present study had three main objectives. First, we sought to extend the CADRI measure to include typical everyday actions and behaviors that could be experienced by adolescents who have been in a romantic relationship. Our second objective was to replicate the multidimensionality of TDV as observed in the other measures using exploratory and confirmatory factor analytical approaches. Finally, we aimed to establish concurrent validity with this measure using participants’ ratings of alcohol overconsumption, cannabis use, and rape myth acceptance views as outcome variables.

## Materials and Methods

### Participants

A total of 730 adolescents between grades 7 and 12 were recruited from high schools in three provinces (i.e., Quebec, Ontario, and Manitoba) in Canada (*M*_age_ = 15.88 years, *SD* = 1.23). Twelve schools participated in the study, four of which were from Quebec, six were from Ontario, and two from Manitoba. Demographic information for the full sample is presented in Table 1. The majority of participants identified their gender as female, followed by male, and non-binary. Half of the sample also identified their ethnicity as White/European (50.1%), and most of the participants did not have previous experiences with relationship violence (90.9%).

**TABLE 1:** Demographic information of samples.

	Sample 1	Sample 2	Total sample
*n*	353	377	730
Age *M* (*SD*)	15.89 (1.23)	15.89 (1.29)	15.89 (1.26)
***Gender***			
Woman	63.7%	62.3%	63.0%
Man	34.8%	34.7%	34.8%
Non-Binary	0.6%	1.6%	1.1%
No answer	0.8%	1.3%	1.1%
***Grade***			
7	0%	0.3%	0.1%
8	0%	0.8%	0.4%
9	26.9%	26.5%	26.7%
10	27.5%	26.3%	26.8%
11	26.9%	26.8%	26.8%
12	17.6%	17.5%	17.5%
No answer	1.1%	1.9%	1.5%
***Ethnicity***			
African/Caribbean	8.2%	11.9%	10.1%
East Asian	6.5%	4.0%	5.2%
First Nations	4.8%	6.4%	5.6%
Inuit	0%	0.5%	0.3%
Latin American	1.4%	2.4%	1.9%
Métis	2.0%	3.2%	2.6%
Middle Eastern/West Asian	3.4%	3.7%	3.6%
South Asian	6.2%	5.8%	6.0%
Southeast Asian	12.5%	15.9%	14.2%
White/European	50.1%	48.0%	49.0%
Different	6.2%	6.9%	6.6%
No answer	14.4%	12.2%	13.3%
***Experiences with relationship violence***			
Yes	7.6%	9.3%	8.5%
No	90.9%	87.8%	89.3%
No answer	1.4%	2.9%	2.2%

*The percentage of ethnicity is greater than 100% because participants could identify with more than one ethnic group.*

### Procedure

Ethical approval was obtained from each of the relevant research ethics bodies at the universities and local school boards. The research team first visited each participating school to explain the purpose of the study and administer consent forms. Once a signed guardian consent form was returned, students were asked to provide assent to participate in the study. During the study, participants responded to various questionnaires that lasted approximately 1 h.

### Measures

#### Teen Dating Aggression Measure

We modified the original CADRI to capture adolescent dating violence more adequately and to expand upon the types of violence assessed. Our modification included combining the two items assessing sexual violence to address potential concerns by schools and their respective boards, i.e., “Forced me to have sex with them when I didn’t want to” and “Touched me sexually when I didn’t want them to” were combined into one broad item: “Forced me to do something sexual that I didn’t want to do.” After the original CADRI items, 20 new statements were added that describe additional forms of violence such as coercion (e.g., “Kept pressuring me to do something even after I made it clear that I did not want to”) and control (e.g., “Made me let them read my emails or texts when I didn’t want them to”). For each of the 44 items on the adapted scale, participants rated how often the behavior occurred with a dating partner over the past three months using a Likert scale from 0 (“*never*”) to 4 (“*most days of the week*”).

#### Rape Myth Acceptance

Participants were asked to rate 10 items that assessed how much they agreed with statements regarding rape myths (RMA) on 4-point Likert scale ranging from 1 (“*Strongly Disagree*”) to 4 (“*strongly agree*”). Items were adapted from established scales of this construct (e.g., Illinois Rape Myth Acceptance Scale; Payne et al., 1999). Consistent with Dworkin et al. (2017), two scores were computed. The first reflected a measure of traditional gender expectations (e.g., “Girls should have sex with the guy they are dating when he wants”) while the second measured rape denial (e.g., “If a girl is sexually assaulted while drunk, she is to blame”). Items were summed for each score and higher scores indicated greater endorsement of RMA. Cronbach’s alpha for traditional gender expectations (0.81) and rape denial (0.73) were high across both samples.

#### Cannabis and Alcohol Consumption

Participants were asked to assess how often they used cannabis or marijuana using a 6-point Likert scale, ranging from 1 (“*Never*”) to 6 (“*About 6 or 7 times a week*”) (*M* = 1.48, *SD* = 1.06). Similarly, alcohol overconsumption in the past month (i.e., drinking to the point of drunkenness) was rated on a 6-point Likert scale, ranging between 1 (“*Never*”) and 6 (“*5 or more times*”) (*M* = 1.22, *SD* = 0.64).

### Plan of Analysis

The analytical plan followed five steps. First, we split the sample using complex sampling procedures (described below). Second, descriptive correlations and inter-item correlations were computed in SPSS to evaluate normality of the data. Third, an EFA of the first sample was conducted. Fourth, CFA to verify the factor structure that was derived from the EFA with the second sample. Finally, bivariate correlations were conducted to examine the associations among the scales and with adolescent rape myth acceptance, and cannabis and alcohol consumption. The EFA and CFA were analyzed with M*plus* 8 (Muthén and Muthén, 1998-2017). To evaluate the fit of the models, fit indices suggested by Hu and Bentler (1999) and Kline (2016), which includes the Comparative Fit Index (CFI; values of at least 0.90), the Tucker-Lewis Index (TLI; values of at least 0.95), the Root Mean Squared Error of Approximation (RMSEA; values no greater than 0.08), and the Weighted Root Mean Squared Error of Approximation (WRMR; values no greater than 1.00) were used.

#### Complex Sampling

In order to examine the factor structure and provide validation, complex random sampling in SPSS was used to split the main sample into two groups. Three strata were used to randomly assign participants into one of the two samples: (a) gender identity, (b) grade level, and (c) previous experience with relationship violence. Sample 1 (S1) was comprised of 353 participants (63.7% female) and Sample 2 (S2) included 377 participants (62.3% female).

## Results

### Descriptive Statistics

Descriptive statistics including skewness and kurtosis were calculated for each item in both samples (see Table 2). In general, each of the items did not have a mean score above 2 but had skewness and kurtosis values that exceeded |1.50| (Tabachnick and Fidell, 2013). Given that these items were rarely endorsed by the participants, the data were treated as categorical rather than continuous, but maintained the original Likert scale options that were originally posed. Moreover, we conducted a missing values analysis and found that none of the items had a proportion of missing values that exceeded 5% (between 2.10 and 3.00%).

**TABLE 2:** Descriptive statistics of items.

	Sample 1			Sample 2		
Item	*M* (*SD*)	Skewness	Kurtosis	*M* (*SD*)	Skewness	Kurtosis
1. Tried to turn my friends against me.	1.14 (0.52)	4.827	26.266	1.08 (0.41)	6.704	52.934
2. Said or did something just to make me feel jealous.	1.47 (0.93)	2.280	4.754	1.30 (0.75)	3.097	10.439
3. Destroyed or threatened to destroy something I valued.	1.06 (0.33)	7.601	71.641	1.08 (0.46)	6.952	51.593
4. Brought up something bad I had done in the past.	1.37 (0.81)	2.605	7.068	1.32 (0.82)	2.982	8.684
5. Threw something at me.	1.06 (0.29)	4.925	25.558	1.06 (0.39)	8.034	69.774
6. Said or did something just to make me angry.	1.43 (0.88)	2.249	4.582	1.30 (0.79)	3.210	10.647
7. Spoke to me in a hostile or mean tone of voice.	1.31 (0.79)	2.963	8.701	1.23 (0.73)	3.869	15.505
8. Forced me to do something sexual that I didn’t want to.	1.10 (0.48)	5.798	37.403	1.11 (0.48)	5.903	39.534
9. Threatened me to get me to do something sexual with him/her.	1.06 (0.38)	8.290	75.200	1.03 (0.27)	9.238	89.141
10. Insulted me.	1.31 (0.74)	2.879	8.722	1.24 (0.71)	3.390	11.834
11. Kissed me when I didn’t want him/her to.	1.12 (0.52)	5.028	27.856	1.11 (0.50)	5.357	31.206
12. Said things to my friends about me to turn them against me.	1.10 (0.48)	5.628	35.374	1.07 (0.36)	6.800	55.719
13. Ridiculed or made fun of me in front of other people.	1.15 (0.50)	4.269	20.880	1.15 (0.58)	5.007	27.423
14. Kept track of who I was with and where I was.	1.46 (1.03)	2.419	4.908	1.32 (0.91)	3.244	9.749
15. Blamed me for a problem or fight we were having.	1.39 (0.90)	2.784	7.463	1.33 (0.84)	3.139	9.986
16. Kicked, hit, or punched me.	1.06 (0.33)	7.636	72.293	1.04 (0.27)	8.701	83.428
17. Accused me of flirting with someone else.	1.38 (0.89)	2.768	7.410	1.27 (0.76)	3.393	11.855
18. Tried to frighten me on purpose.	1.11 (0.46)	5.005	28.453	1.10 (0.48)	5.724	35.086
19. Slapped me or pulled my hair.	1.10 (0.43)	6.212	46.008	1.06 (0.35)	6.710	47.969
20. Threatened to hurt me.	1.04 (0.26)	7.739	68.247	1.04 (0.32)	9.080	92.644
21. Threatened to break up with me or end our friendship.	1.15 (0.52)	4.866	28.858	1.16 (0.64)	4.757	23.674
22. Threatened to hit or throw something at me.	1.03 (0.25)	10.331	115.005	1.03 (0.27)	10.823	135.353
23. Pushed, shoved, grabbed, or shook me.	1.07 (0.32)	6.146	44.956	1.08 (0.38)	5.768	41.353
24. Spread rumors about me.	1.11 (0.51)	5.754	35.926	1.11 (0.43)	4.357	19.650
25. Screamed or yelled at me.	1.18 (0.60)	4.298	21.027	1.16 (0.60)	4.648	23.063
26. Said mean things to me.	1.31 (0.75)	2.893	8.835	1.27 (0.77)	3.617	13.527
27. Left me out of an activity or a social group on purpose.	1.11 (0.46)	5.836	39.436	1.12 (0.51)	5.596	34.564
28. Told me that he/she would break up with me or end our friendship if I did not do something he/she wanted.	1.11 (0.53)	5.827	36.527	1.16 (0.60)	5.407	29.007
29. Said means things about me to other people.	1.12 (0.48)	5.401	34.067	1.27 (0.77)	4.386	20.275
30. Talked about how other people were better or more fun than me.	1.15 (0.58)	4.911	26.171	1.14 (0.57)	4.737	24.246
31. Told me that other people didn’t like me.	1.16 (0.59)	4.458	21.801	1.13 (0.51)	4.749	25.825
32. Told me that I was not a good boyfriend/girlfriend or friend.	1.13 (0.51)	5.025	29.018	1.16 (0.62)	4.759	23.904
33. Gave me the silent treatment.	1.35 (0.76)	2.489	6.015	1.31 (0.78)	3.051	9.504
34. Got upset when I spent time with other people.	1.48 (1.01)	2.280	4.387	1.31 (0.82)	3.208	10.342
35. Said mean things to me about someone else who is important to me.	1.25 (0.73)	3.393	11.941	1.22 (0.72)	3.958	16.238
36. Got upset when I did really well on something.	1.09 (0.43)	6.006	39.999	1.05 (0.35)	9.136	95.263
37. Told me that I needed to spend more time with him/her.	1.47 (1.04)	2.321	4.374	1.33 (0.81)	3.023	9.474
38. Made me let them read my e-mails or texts when I didn’t want them to.	1.18 (0.65)	4.132	17.189	1.11 (0.56)	5.937	36.925
39. Made me do something I really didn’t want to do.	1.13 (0.55)	5.237	30.363	1.11 (0.52)	5.966	38.681
40. Was mean to me or insulted me to get me to do something for him/her.	1.10 (0.49)	5.958	38.831	1.09 (0.51)	6.746	47.175
41. Got mad at me when I said “no” to him/her about something.	1.25 (0.75)	3.525	12.582	1.20 (0.64)	4.214	19.880
42. Threatened me to try to get me to do something he/she wanted me to do.	1.10 (0.52)	6.180	40.223	1.07 (0.42)	7.353	58.502
43. Insulted me or said mean things to me when I said “no” to him/her about doing something.	1.14 (0.61)	5.129	27.284	1.09 (0.51)	6.593	46.002
44. Kept pressuring me to do something even after I made it clear that I did not want to.	1.22 (0.75)	3.762	13.993	1.17 (0.65)	4.734	23.186

### Exploratory Factor Analysis

An EFA was conducted on the first sample using the weighted least squares mean and variance adjusted (WLSMV) estimator and the geomin oblique rotation (epsilon = 0.50). Using the eigenvalue-greater-than-1 rule (Kaiser, 1960), results from this analysis suggest a 7-factor model (see Table 3). However, the increase in number of factors could also result in multiple cross-loadings, therefore, we also examined the changes in model fit to determine which factor structure best fit the data. Specifically, we calculated the change (Δ) in RMSEA, CFI, TLI, and SRMR by comparing each model with the model that preceded it. For example, the change in fit for a 2-factor solution was calculated by taking the difference between its fit and the fit from the 1-factor solution. In line with empirical suggestions for factor retention (e.g., Clark and Bowles, 2018), ΔCFI and ΔTLI improvements of at least 0.01 were considered in determining the final factor structure.

**TABLE 3:** Exploratory factor analysis (EFA) model fit indices for the factor structure of the Teen Dating Aggression Measure (TeDAM).

			Chi-Square test of model fit										
Solution	Eigenvalue	Δ	χ^2^	*df*	*p*	RMSEA	Δ	CFI	Δ	TLI	Δ	SRMR	Δ
1-Factor	25.46	0.71	1109.419	740	<0.001	0.038	–	0.972	–	0.971	–	0.112	–
2-Factor	3.051	0.08	897.463	701	<0.001	0.028	0.010	0.985	0.013	0.984	0.013	0.082	0.03
3-Factor	2.052	0.06	774.471	663	0.002	0.022	0.006	0.992	0.007	0.99	0.006	0.068	0.014
4-Factor	1.625	0.05	697.42	626	0.025	0.018	0.004	0.995	0.003	0.993	0.003	0.057	0.011
5-Factor	1.124	0.03	638.449	590	0.082	0.015	0.003	0.996	0.001	0.995	0.002	0.052	0.005
6-Factor	1.064	0.03	579.466	555	0.229	0.011	0.004	0.998	0.002	0.997	0.002	0.047	0.005
7-Factor	0.899	0.02	530.392	521	0.378	0.007	0.004	0.999	0.001	0.999	0.002	0.043	0.004

*Analysis was performed on Sample 1 and items 13, 18, 28, and 43 were removed. All chi-square tests were statistically significant, p < 0.05. Δ represents the change between for each respective fit index from the current model and a previous model.*

Upon examination of change in model fit as well as considering the overall conceptualization of the factors, a 3-factor solution appeared to be the best fitting model (see Table 3). In this model, the change in model fit from a 2-factor solution to a 3-factor solution produced ΔRMSEA = 0.02, ΔCFI = 0.01, ΔTLI = 0.01, and ΔSRMR = 0.01. The change in the subsequent models did not necessarily improve the overall model fit. From there, the factor loadings of the items were evaluated; items that cross-loaded onto more than one factor were dropped if the standardized loading was < 0.32. The correlations between each of the three factors (F1, F2, F3) were statistically significant (*p* < 0.05): F1-F2 = 0.46, F1-F3 = 0.52, F2 and F3 = 0.50.

Further investigation of the items showed that three items closely cross-loaded (difference between 0.02 and 0.06) onto two factors and did not quantitatively or conceptually fit with the factor in which it strongly loaded (discussed next). The three items were: (a) “Ridiculed or made fun of me in front of other people”; (b) “Told me that he/she would break up with me if I did not do something he/she wanted”; and (c) “Insulted me or said mean things to me when I said ‘no’ to him/her about doing something.” Thus, we removed the items and reanalyzed the EFA, which still supported a 3-factor solution. Table 3 provides fit indices and change (Δ) in model fit between each of the solutions. In this final model, there were fewer cross-loadings; when they did occur, there was discussion of the item in question and consensus was achieved if the authors agreed that it conceptually fit with the factor to which it was primarily loaded. The three factors consisted of constructs related to: (a) psychological aggression, (b) physical, sexual aggression, and (c) relational aggression. Table 4 provides the standardized loadings for each of the three factors.

**TABLE 4:** Standardized factor loadings and descriptive statistics for the 3-factor solution of the Teen Dating Aggression Measure (TeDAM).

	3-Factor solution		
Item	F1	F2	F3
**F1: Psychological aggression**			
1. Tried to turn my friends against me.	0.37*		
2. Said or did something just to make me feel jealous.	0.58*		
3. Destroyed or threatened to destroy something I valued.	0.77*		
4. Brought up something bad I had done in the past.	0.74*		
6. Said or did something just to make me angry.	0.71*		
7. Spoke to me in a hostile or mean tone of voice.	0.63*		0.38*
10. Insulted me.	0.64*		
14. Kept track of who I was with and where I was.	0.76*		
15. Blamed me for a problem or fight we were having.	0.64*		0.40*
17. Accused me of flirting with someone else.	0.82*		
25. Screamed or yelled at me.	0.62*		
26. Said mean things to me.	0.52*		0.46*
33. Gave me the silent treatment.	0.54*		
34. Got upset when I spent time with other people.	0.72*		
35. Said mean things to me about someone else who is important to me.	0.42*	0.34*	
37. Told me that I needed to spend more time with him/her.	0.59*	0.45*	
38. Made me let them read my e-mails or texts when I didn’t want them to.	0.65*	0.43*	
**F2: Physical, sexual aggression**			
5. Threw something at me.		0.42*	
8. Forced me to do something sexual that I didn’t want to.		0.80*	
9. Threatened me to get me to do something sexual with him/her.		0.82*	
11. Kissed me when I didn’t want him/her to.	0.45*	0.61*	
16. Kicked, hit, or punched me.		0.74*	
19. Slapped me or pulled my hair.		0.61*	
20. Threatened to hurt me.		0.64*	0.34*
22. Threatened to hit or throw something at me.		0.86*	
23. Pushed, shoved, grabbed, or shook me.		0.87*	
39. Made me do something I really didn’t want to do.		0.58*	
40. Was mean to me or insulted me to get me to do something for him/her.		0.67*	0.40*
41. Got mad at me when I said “no” to him/her about something.	0.33*	0.56*	
42. Threatened me to try to get me to do something he/she wanted me to do.		0.65*	0.41*
44. Kept pressuring me to do something even after I made it clear that I did not want to.		0.67*	
**F3: Relational aggression**			
12. Said things to my friends about me to turn them against me.			0.65*
21. Threatened to break up with me or end our friendship.	0.39*		0.65*
24. Spread rumors about me.			0.86*
27. Left me out of an activity or a social group on purpose.			0.83*
29. Said means things about me to other people.			0.86*
30. Talked about how other people were better or more fun than me.			0.53*
31. Told me that other people didn’t like me.			0.65*
32. Told me that I was not a good boyfriend/girlfriend			0.63*
36. Got upset when I did really well on something.		0.38*	0.62*

Analysis was performed on Sample 1 and items 13, 18, 28, and 43 were removed. All factor loadings were statistically significant, *p < 0.05.

#### Factor 1: Psychological Aggression

The first factor was comprised of 17 items, with standardized loadings ranging from 0.37 to 0.82. These items appear to reflect a psychological or manipulative form of aggression perpetrated to victims. Example items include, “gave me the silent treatment,” “insulted me,” “brought up something bad I had done in the past,” and “accused me of flirting with someone else.” The Cronbach’s alpha for this scale was 0.94.

#### Factor 2: Physical, Sexual Aggression

In the second factor, the standardized loadings of 14 items (factor loadings ranging between 0.42 and 0.87) suggest overt physical and sexual aggression acts committed toward a victim. Example items within this factor include, “threw something at me,” “threatened to hurt me,” “kicked, hit, or punched me,” and “kissed me when I didn’t want him/her to.” Cronbach’s alpha was 0.89.

#### Factor 3: Relational Aggression

In this last factor, 9 items with standardized loadings ranging between 0.53 and 0.86 reflect an indirectly applied form of relational aggression. In particular, the items reflected behaviors that a partner may do or say to others about a victim. Unlike the first factor that focused on psychological aggression targeted toward a victim, the items on this scale generally involved behaviors that implicated other individuals, such as a victims’ friends. Some examples from this scale include, “talked about how other people were better or more fun than me,” “left me out of an activity or social group on purpose,” “spread rumors about me,” and “said mean things about me to other people.” Cronbach’s alpha for this scale was 0.91.

### Confirmatory Factor Analysis

Three separate CFAs were conducted with the second sample to compare a 3-factor model with a 2-factor model and 1-factor model, respectively. Given the use of the WLSMV estimator, the DIFFTEST function was employed using the 3-factor model as the initial comparison against the 2-factor and 1-factor models. In comparison to the 3-factor model, the 2-factor and 1-factor models each had statistically significant worse fit (all *p*s < 0.001). Thus, we retained the 3-factor model identified from the EFA used with the first sample (see Table 5). In general, model fit was acceptable, χ^2^(737) = 1611.27, *p* < 0.001, CFI = 0.94, RMSEA = 0.06 (0.05–0.06); standardized factor loadings were strong (>0.60) and statistically significant (*p* < 0.001) (see Table 6 for loadings by factor). Further, reliability using McDonald’s omega (ω), which does not assume equal factor loadings (see Hayes and Coutts, 2020), showed that the scales were highly reliable.

**TABLE 5:** Confirmatory factor analysis (CFA) model fit for the factor structure of the Teen Dating Aggression Measure (TeDAM).

	Chi-Square test of model fit											Chi-Square for difference test		
Solution	χ^2^	*df*	*p*	RMSEA	Δ	CFI	Δ	TLI	Δ	WRMR	Δ	χ^2^	*df*	*p*
3-Factor	1611.267	737	<0.001	0.057	0.000	0.941	0.001	0.938	0.002	1.588	0.019	–	–	–
2-Factor	1633.886	739	<0.001	0.057	0.000	0.940	0.001	0.936	0.000	1.607	0.009	36.069	2	<0.001
1-Factor	1644.878	740	<0.001	0.057	–	0.939	–	0.936	–	1.616	–	42.621	3	<0.001

*Analysis was performed on Sample 2 and items 13, 18, 28, and 43 were removed All chi-square tests were statistically significant. Difference test uses the DIFFTEST option in Mplus using the 3-factor solution as the initial comparison (it has more free parameters). Statistically significant chi-square for difference tests mean that adding more restrictions (i.e., 2-factor and 1-factor) worsens model fit.*

**TABLE 6:** Confirmatory factor analysis standardized loadings and omega reliability estimates (ω).

	3-Factor solution			
Item	ω	F1	F2	F3
**F1: Psychological aggression**	0.98			
1. Tried to turn my friends against me.		0.85*		
2. Said or did something just to make me feel jealous.		0.84*		
3. Destroyed or threatened to destroy something I valued.		0.89*		
4. Brought up something bad I had done in the past.		0.86*		
6. Said or did something just to make me angry.		0.89*		
7. Spoke to me in a hostile or mean tone of voice.		0.89*		
10. Insulted me.		0.82*		
14. Kept track of who I was with and where I was.		0.80*		
15. Blamed me for a problem or fight we were having.		0.89*		
17. Accused me of flirting with someone else.		0.82*		
25. Screamed or yelled at me.		0.86*		
26. Said mean things to me.		0.92*		
33. Gave me the silent treatment.		0.84*		
34. Got upset when I spent time with other people.		0.90*		
35. Said mean things to me about someone else who is important to me.		0.84*		
37. Told me that I needed to spend more time with him/her.		0.78*		
38. Made me let them read my e-mails or texts when I didn’t want them to.		0.89*		
**F2: Physical, sexual aggression**	0.98			
5. Threw something at me.			0.73*	
8. Forced me to do something sexual that I didn’t want to.			0.87*	
9. Threatened me to get me to do something sexual with him/her.			0.78*	
11. Kissed me when I didn’t want him/her to.			0.77*	
16. Kicked, hit, or punched me.			0.77*	
19. Slapped me or pulled my hair.			0.90*	
20. Threatened to hurt me.			0.87*	
22. Threatened to hit or throw something at me.			0.85*	
23. Pushed, shoved, grabbed, or shook me.			0.87*	
39. Made me do something I really didn’t want to do.			0.95*	
40. Was mean to me or insulted me to get me to do something for him/her.			0.97*	
41. Got mad at me when I said “no” to him/her about something.			0.91*	
42. Threatened me to try to get me to do something he/she wanted me to do.			0.95*	
44. Kept pressuring me to do something even after I made it clear that I did not want to.			0.91*	
**F3: Relational aggression**	0.96			
12. Said things to my friends about me to turn them against me.				0.82*
21. Threatened to break up with me or end our friendship.				0.84*
24. Spread rumors about me.				0.88*
27. Left me out of an activity or a social group on purpose.				0.62*
29. Said means things about me to other people.				0.86*
30. Talked about how other people were better or more fun than me.				0.84*
31. Told me that other people didn’t like me.				0.94*
32. Told me that I was not a good boyfriend/girlfriend				0.91*
36. Got upset when I did really well on something.				0.80*

Analysis was performed on Sample 2 and items 13, 18, 28, and 43 were removed. All factor loadings were statistically significant, *p < 0.05.

### Mean-Level Differences by Gender Identity and Grade Level

Scores for each of the three dimensions were aggregated for each of the three scales and then compared as a function of participant gender identity and grade level. A one-way MANOVA with the three scores as the dependent variables did not yield a statistically significant multivariate effect of gender identity for Sample 1, Wilk’s λ = 0.97, *F*(9, 825.19) = 1.31, *p* = 0.23, η*_*p*_*^2^ = 0.01, or Sample 2, Wilk’s λ = 0.97, *F*(9, 886.03) = 1.28, *p* = 0.24, η*_*p*_*^2^ = 0.01. Additionally, there were no observed multivariate effects of grade level for Sample 1, Wilk’s λ = 0.98, *F*(12, 894.44) = 0.52, *p* = 0.90, η*_*p*_*^2^ = 0.01, or Sample 2, Wilk’s λ = 0.95, *F*(18, 1021.55) = 1.00, *p* = 0.46, η*_*p*_*^2^ = 0.02. The lack of significant group differences suggest that the means from each of the three dimensions did not significantly differ as a function of the participant’s gender identity or their grade.

### Concurrent Validity

In the last analysis, we tested concurrent associations between the TeDAM, perceptions of rape myths acceptance, cannabis use, and alcohol consumption (see Table 7 for descriptive statistics). Accordingly, bivariate correlations including bootstrapping (*n* = 10,000) to estimate 95% confidence intervals were computed between the mean scores of the three factors along with cannabis use, alcohol overconsumption, and rape myth acceptance for the whole sample. Results showed similar findings across each of the three factors (see Table 8). Psychological aggression was positively associated with alcohol overconsumption, cannabis use, and rape denial. Physical and sexual aggression was also positively correlated with each of alcohol overconsumption, cannabis use, and rape denial. Similarly, relational aggression was positively related to alcohol overconsumption, cannabis use, and rape denial. There were no statistically significant associations between the TeDAM scales and traditional gender expectations (*p*s > 0.05).

**TABLE 7:** Descriptive statistics of study variables for the overall sample.

Variable	*M* (*SD*)	Minimum	Maximum
**TeDAM**			
Psychological aggression	1.29 (0.56)	1.00	4.28
Physical, sexual aggression	1.10 (0.32)	1.00	4.41
Relational aggression	1.12 (0.38)	1.00	4.89
Cannabis use	1.48 (1.06)	1.00	6.00
Alcohol overconsumption	1.22 (0.64)	1.00	6.00
**Rape myth acceptance**			
Traditional gender expectations	3.82 (1.40)	3.00	12.00
Rape denial	16.00 (6.80)	4.00	16.00

*Scores on the TeDAM are an average of the number of items for each scale, while the rape myth acceptance scores are summed items.*

**TABLE 8:** Bivariate correlations between Teen Dating Aggression Measure (TeDAM) scales and outcome variables.

	Cannabis use	Alcohol overconsumption	RMA—Traditional gender expectations	RMA rape denial
Psychological aggression	0.28** (0.16–0.39)	0.17* (0.06–0.30)	0.02 (–0.06 to 0.16)	0.10* (0.01–0.21)
Physical, sexual aggression	0.21** (0.11–0.32)	0.12* (0.01–0.25)	0.03 (–0.10 to –0.21)	0.11* (–0.02 to 0.23)
Relational aggression	0.23** (0.11–0.35)	0.17** (0.11–0.35)	0.02 (–0.09 to –0.17)	0.08* (–0.03 to 0.20)

95% confidence intervals are reported with bootstrapping = 10,000. RMA, rape myth acceptance scale. *p < 0.05; **p < 0.001.

## Discussion

Findings from this study provide initial psychometric support for the TeDAM and its use among adolescents. Specifically, factor analyses for the TeDAM suggested that a solution that included three factors, namely behaviors regarding (a) psychological aggression, (b) physical and sexual aggression, and (c) relational aggression, were most appropriate. This factor structure was further supported in a CFA and high reliability estimates.

Each of the factors represented the various ways in which adolescents might experience dating aggression. The first factor, physical and sexual aggression, is common among all measures within the TDV literature. In line with other measures of dating violence victimization, including the CADRI (Wolfe et al., 2001) and the MARSHA (Rothman et al., 2021), the TeDAM yielded a factor that incorporated elements of physical (e.g., slapped, hair pulled, objects thrown) as well as sexual aggression (e.g., threats to coerce sexual acts, sexual force). The second factor reflected psychological aggression, which are harmful verbal and/or emotional acts directed toward the victim (Follingstad et al., 2005). Previous work by Follingstad (2007) suggests that psychological aggression in the form of humiliation and insults differs from physically aggressive acts. Indeed, the factor structure of the TeDAM supports the claim that dating violence and aggression should not be limited to physical acts. Specifically, psychological aggression included items related to verbal and emotional aggression (e.g., making accusations, insulting, giving the silent treatment) as well as manipulative forms of aggression (e.g., made to allow to read social media, keeping track of a partner). Lastly, the third factor related to acts that were characterized as relational aggression. Although this factor could be interpreted similarly to the psychological aggression, the relational component referred to behaviors pertaining to the implicates the peer group (or others) to harm the relationship. For example, items within this scale included aggressive acts such as spreading rumors or using others (e.g., a victim’s friends) to make threats or exclude a partner. Within the context of adolescent peer relations, psychological forms of aggression are distinct from relational aggression (see Linder et al., 2002). In particular, the target of psychological aggression is the victim while the target for relational aggression is the relationship. The methods employed within each form of aggression can vary, but the target remains consistent. The addition of a scale focused on relational aggression scale is supported by previous research on IPV, in which romantic relational victimization was found to be negatively correlated with romantic relationship quality (Linder et al., 2002).

There were three findings related to the validation of the measure. First, we found that each of the three factors was associated with adolescent cannabis use and the overconsumption of alcohol. Specifically, more frequent experiences with each of the three aggressive factors were associated with increased use of cannabis and drinking. These findings are in line with previous work that suggests that cannabis and alcohol are associated with victimization from TDV and assault (e.g., Dworkin et al., 2017; Johnson et al., 2017). The last two findings refer to the association between the TDV factors and rape myth acceptance. In particular, there were no significant associations with the traditional gender expectations scale, whereas each of the factors were positively correlated with rape denial. Previous studies have shown positive associations between rape myth acceptance and sexual dating aggression (Reyes and Foshee, 2013). Our findings are partially in line with these results, with the exception of traditional gender expectations. There are two potential methodological explanations for this. First, the IRMAS, the measure with which the items were derived and adapted, is known to produce floor effects, which could explain the lack of a significant association (Trottier et al., 2021). Moreover, with the exception of Dworkin et al. (2017), research on the association between TDV victimization and rape myth acceptance and its specific subscales have not been investigated with an adolescent population and therefore requires replication.

Broadly, the strengths of the TeDAM include the support for a scale on relational aggression. This addition is important because it incorporates the typical actions and behaviors that adolescents engage in with their peers as well as their romantic partners, thus making the measure more developmentally relevant. From a measurement perspective, the TeDAM is also straightforward to use, easy to score, and has strong reliability and validity. Nevertheless, this study acknowledges some limitations. First, although the large sample was obtained from three different provinces across Canada, these were not nationally representative, therefore we could not test for sociodemographic differences (e.g., ethnicity). Second, although our sample size was large enough to assess the factor structure of this measure, we were unable to fully evaluate the psychometric properties using measurement invariance, which would address the extent to which the items were interpreted in a similar manner across different groups. As such, replication of the factor structures and an analysis of the equivalence across sex, gender identity, and previous experiences with relationship violence would benefit the utility of the TeDAM. Finally, the present study was focused on dating victimization experiences of youth. However, it is also necessary to consider the extent to which dating partners perpetrate such acts. Given that the additional items were written similarly to the CADRI, we argue that the target of scale can change between the victim to the perpetrator, which would be in line with other measures, including the CADRI (Wolfe et al., 2001) and the MARSHA (Rothman et al., 2021, 2020). We speculate that a similar factor structure would emerge with dating violence perpetrators.

In summary, the goal of this study was to create an assessment of TDV that was designed to reflect the adolescent experience and include relevant forms of TDV. This measure begins to address the gaps in extant TDV measures by including items related to overt sexual violence, psychological aggression, and relationally aggressive behaviors. Results provided initial psychometric support for a developmentally relevant assessment of adolescent aggressive experiences in the context of romantic relationships. Given both the short- and long-term consequences of victimization from dating violence, there are meaningful implications for researchers, as this could provide more authentic findings when investigating the phenomenon of TDV, as well as have important implications for practitioners (e.g., clinicians) looking to obtain a more comprehensive view of the experiences of TDV victimization among the youth they service. Together, with further development and implementation of the TeDAM, this study has crucial theoretical implications as it could help increase our understanding in the field of TDV.

## Data Availability Statement

The datasets presented in this article are not readily available because there are ethical and consent restrictions that do not allow for the release of data outside of the research team. Requests to access the datasets should be directed to WC, wendy.craig@queensu.ca.

## Ethics Statement

The studies involving human participants were reviewed and approved by 1. Research Ethics Board Office (McGill University) #20-07-044 - Research Ethics, 2. General Research Ethics Board (Queen’s University) #GPSYC-997-20. Written informed consent to participate in this study was provided by the participants’ legal guardian/next of kin.

## Author Contributions

WC, MD, AM-S, and CK conceptualized and designed the larger study which includes this study. ND coordinated data collection as well as cleaned the data. For this manuscript, RP, TW, LV-M, and CK contributed to the conceptualization and plan. RP organized the data. RP and LV-M conducted the statistical analyses. RP wrote the first draft of the manuscript. TW, LV-M, and ND wrote sections of the manuscript. CK oversaw the manuscript writing throughout the process. All authors contributed to manuscript revision, read, and approved the submitted version.

## Conflict of Interest

The authors declare that the research was conducted in the absence of any commercial or financial relationships that could be construed as a potential conflict of interest.

## Publisher’s Note

All claims expressed in this article are solely those of the authors and do not necessarily represent those of their affiliated organizations, or those of the publisher, the editors and the reviewers. Any product that may be evaluated in this article, or claim that may be made by its manufacturer, is not guaranteed or endorsed by the publisher.

## References

[B1] Asghari M. Connolly J. Cochrane-Brink K. (2021). Peer and dating aggression among early adolescent boys and girls admitted to a secure inpatient psychiatric unit: links with maltreatment. *J. Aggress. Maltrea. Trauma* 30, 154–174. 10.1080/10926771.2020.1783735

[B2] Alleyne B. Coleman-Cowger V. H. Crown L. Gibbons M. A. Vines L. N. (2011). The effects of dating violence, substance use and risky sexual behavior among a diverse sample of Illinois youth. *J. Adolesc.* 34 11–18. 10.1016/j.adolescence.2010.03.006 20452662

[B3] Breiding M. Basile K. C. Smith S. G. Black M. C. Mahendra R. R. (2015). *Intimate Partner Violence Surveillance: Uniform Definitions and Recommended Data Elements. Version 2.0.* Atlanta, GA: National Center for Injury Prevention and Control.

[B4] Cascardi M. Avery-Leaf S. O’Leary K. D. Slep A. M. S. (1999). Factor structure and convergent validity of the Conflict Tactics Scale in high school students. *Psychol. Assess.* 11 546–555. 10.1037/1040-3590.11.4.546

[B5] Centers for Disease Control and Prevention (2020). *Preventing Teen Dating Violence.* Available online at: https://www.cdc.gov/violenceprevention/intimatepartnerviolence/teendatingviolence/fastfact.html (accessed December 30, 2020).

[B6] Clark D. A. Bowles R. P. (2018). Model fit and item factor analysis: overfactoring, underfactoring, and a program to guide interpretation. *Multivariate Behav. Res.* 53 544–558. 10.10180/00273171.2018.1461058 29683723

[B7] Collins W. A. Welsh D. P. Furman W. (2009). Adolescent romantic relationships. *Annu. Rev. Psychol.* 60 631–652. 19035830 10.1146/annurev.psych.60.110707.163459

[B8] Dosil M. Jaureguizar J. Bernaras E. Sbicigo J. B. (2020). Teen dating violence, sexism, and resilience: a multivariate analysis. *Int. J. Environ. Res. Public Health* 17, 1–18. 10.3390/ijerph17082652 32294915 PMC7215810

[B9] Dworkin E. R. Sessarego S. N. Pittenger S. L. Edwards K. M. Banyard V. (2017). Rape myth acceptance in sexually-assaulted adolescents’ school contexts: associations with depressed mood and alcohol use. *Am. J. Community Psychol.* 60 516–526. 10.1002/ajcp.12173 28921576 PMC5830101

[B10] Edwards K. M. Turchik J. A. Dardis C. M. Reynolds N. Gidycz C. A. (2011). Rape myths: history, individual and institutional-level presence, and implications for change. *Sex Roles* 65 761–773. 10.1007/s11199-011-9943-2

[B11] Ellis W. E. Crooks C. V. Wolfe D. A. (2009). Relational aggression in peer and dating relationships: links to psychological and behavioural adjustment. *Soc. Dev*. 18, 253–269. 10.1111/j.1467-9507.2008.00468.x

[B12] Espelage D. L. Leemis R. W. Niolon P. H. Kearns M. Basile K. C. Davis J. P. (2020). Teen dating violence perpetration: protective factor trajectories from middle to high school among adolescents. *J. Res. Adolesc.* 30 170–188. 10.1111/jora.12510 31169951 PMC6895399

[B13] Exner-Cortens D. (2018). “Measuring adolescent dating violence,” in *Adolescent Dating Violence: Theory, Research, and Prevention*, eds Wolfe D. A. Temple J. R. (Cambridge, MA: Academic Press), 315–340. 10.1016/B978-0-12-811797-2.00013-X

[B14] Exner-Cortens D. Baker E. Craig W. (2021). The national prevalence of adolescent dating violence in Canada. *J. Adolescent Health* 69, 495–502. 10.1016/j.adolhealth.2021.01.032 33762133

[B15] Exner-Cortens D. Eckenrode J. Rothman E. (2013). Longitudinal associations between teen dating violence victimization and adverse health outcomes. *Pediatrics* 131 71–78. 10.1542/peds.2012-1029 23230075 PMC3529947

[B16] Exner-Cortens D. Gill L. Eckenrode J. (2016a). Measurement of adolescent dating violence: a comprehensive review (Part 1, behaviours). *Aggress. Violent Behav.* 27 64–78. 10.1016/j.avb.2016.02.007

[B17] Exner-Cortens D. Gill L. Eckenrode J. (2016b). Measurement of adolescent dating violence: a comprehensive review (Part 2, attitudes). *Aggress. Violent Behav.* 27 93–106.

[B18] Fernández-Fuertes A. A. Fuertes A. Pulido R. F. (2006). Evaluación de la violencia en las relaciones de pareja de los adolescentes. Validación del Conflict in Adolescent Dating Relationships Inventory (CADRI)-versión española. *Int. J. Clin. Health Psychol.* 6 339–358.

[B19] Fernández-González L. Wekerle C. Goldstein A. L. (2012). Measuring adolescent dating violence: development of ‘conflict in adolescent dating relationships inventory’ short form. *Adv. Ment. Health* 11 35–54. 10.5172/jamh.2012.11.1.35

[B20] Follingstad D. R. (2007). Rethinking current approaches to psychological abuse: conceptual and methodological issues. *Aggress. Violent Behav.* 12 439–458. 10.1016/j.avb.2006.07.004

[B21] Follingstad D. R. Coyne S. Gambone L. (2005). A representative measure of psychological aggression and its severity. *Violence Vict.* 20 25–38. 10.1891/vivi.2005.20.1.25 16047933

[B22] Furman W. Collins W. (2009). “Adolescent romantic relationships and experiences,” in *Handbook of Peer Interactions, Relationships, and Groups*, eds Rubin K. H. Bukowski W. M. Laursen B. (New York, NY: Guilford Press), 341–360.

[B23] Garthe R. C. Sullivan T. N. Behrhorst K. L. (2021). A latent class analysis of early adolescent peer and dating violence: associations with symptoms of depression and anxiety. *J. Interpers. Violence* 36(5-6), 2031–2049. 10.1177/0886260518759654 29475422

[B24] Goncy E. A. Sullivan T. N. Farrell A. D. Mehari K. R. Garthe R. C. (2017). Identification of patterns of dating aggression and victimization among urban early adolescents and their relations to mental health symptoms. *Psychol. Violence* 7, 58–68. 10.1037/vio0000039

[B25] Hayes A. F. Coutts J. J. (2020). Use omega rather than Cronbach’s alpha for estimating reliability. *But…. Commun. Methods Meas.* 14 1–24. 10.1080/19312458.2020.1718629

[B26] Hokoda A. Ramos-Lira L. Celaya P. Vilhauer K. Angeles M. Ruíz S. (2006). Reliability of translated measures assessing dating violence among Mexican adolescents. *Violence Vict.* 21 117–127. 10.1891/0886-6708.21.1.117 16494137

[B27] Hu L. Bentler P. M. (1999). Cutoff criteria for fit indexes in covariance structure analysis: conventional criteria versus new alternatives. *Struct. Equ. Model.* 6 1–55. 10.1080/10705519909540118

[B28] Johnson R. M. LaValley M. Schneider K. E. Musci R. J. Pettoruto K. Rothman E. F. (2017). Marijuana use and physical dating violence among adolescents and emerging adults: a systematic review and meta-analysis. *Drug Alcohol Depend.* 174 47–57. 10.1016/j.drugalcdep.2017.01.012 28314193 PMC5521998

[B29] Jouriles E. N. Garrido E. Rosenfield D. McDonald R. (2009). Experiences of psychological and physical aggression in adolescent romantic relationships: links to psychological distress. *Child Abuse Negl*. 33, 451–460. 10.1016/j.chiabu.208.11.005 19589597 PMC3951513

[B30] Kaiser H. F. (1960). The application of electronic computers to factor analysis. *Educ. Psychol. Meas.* 20 141–151. 10.1177/001316446002000116

[B31] Kline R. B. (2016). *Principles and Practice of Structural Equation Modeling.* New York, NY: Guilford.

[B32] Knox L. Lomonaco C. Alpert E. (2009). “Adolescent relationship violence,” in *Intimate Partner Violence: A Health-Based Perspective*, eds Mitchell C. Anglin D. (Oxford: Oxford University Press), 511–530.

[B33] Leadbeater B. J. Banister E. M. Ellis W. E. Yeung R. (2008). Victimization and relational aggression in adolescent romantic relationships: The influence of parental and peer behaviours, and individual adjustment. *J. Youth Adolesc*. 37, 359–372. 10.1007/s10964-007-9269-0 27307651 PMC4905751

[B34] Linder J. R. Crick N. R. Collins W. A. (2002). Relational aggression and victimization in young adults’ romantic relationships: associations with perceptions of parent, peer, and romantic relationship quality. *Soc. Dev.* 11 69–86. 10.1111/1467-9507.00187

[B35] Morelli M. Bianchi D. Chirumbolo A. Baiocco R. (2018). The cyber dating violence inventory. Validation of a new scale for online perpetration and victimization among dating partners. *Eur. J. Dev. Psychol*. 15, 464–471. 10.1080/17405629.2017.1305885

[B36] Muthén L. K. Muthén B. O. (1998-2017). *Mplus user’s Guide*, 8th Edn. Los Angeles, CA: Muthén & Muthén.

[B37] Parker E. M. Debnam K. Pas E. T. Bradshaw C. P. (2016). Exploring the link between alcohol and marijuana use and teen dating violence victimization among high school students. The influence of school context. *Health Educ. Behav.* 43 528–536. 10.1177/1090198115605308 26377526 PMC6414043

[B38] Payne D. L. Lonsway K. A. Fitzgerald L. F. (1999). Rape myth acceptance: exploration of its structure and its measurement using the Illinois Rape Myth Acceptance Scale. *J. Res. Pers*. 33, 27–68. 10.1006/jrpe.1998.2238

[B39] Reyes H. L. M. N. Foshee V. A. (2013). Sexual dating aggression across grades 8 through 12: timing and predictors of onset. *J. Youth Adolesc.* 42 581–595. 10.1007/s10964-012-9864-6 23180071 PMC3596483

[B40] Rothman E. F. Cuevas C. A. Mumford E. A. Bahrami E. Taylor B. G. (2021). The psychometric properties of the Measure of Adolescent Relationship Harassment and Abuse (MARSHA) with a nationally representative sample of US youth. *J. Interpers. Violence* 10.1177/0886260520985480 [Epub ahead of print]. 33399026

[B41] Rothman E. F. Paruk J. Cuevas C. A. Temple J. R. Gonzales K. (2020). The development of the Measure of Adolescent Relationship Harassment and Abuse (MARSHA): input from Black and multiracial, Latinx, Native American, and LGBTQ+ youth. *J. Interpers. Violence* 1–24. 10.1177/0886260520936367 32627640

[B42] Schad M. M. Szwedo D. E. Antonishak J. Hare A. Allen J. P. (2008). The broader context of relational aggression in adolescent romantic relationships: predictions from peer pressure and links to psychosocial functioning. *J. Youth Adolesc*. 37, 346–358. 10.1007/s10964-007-9226-y 18523685 PMC2409192

[B43] Shaffer C. S. Adjei J. Viljoen J. L. Douglas K. S. Saewyc E. M. (2021). Ten-year trends in physical dating violence victimization among adolescent boys and girls in British Columbia, Canada. *J. Interpers. Violence* 36(9-10), 3947–3964. 10.1177/0886260518788367 30019602

[B44] Smith J. Mulford C. Latzman N. E. Tharp A. T. Niolon P. H. Blachman-Demner D. (2015). Taking stock of behavioral measures of adolescent dating violence. *J. Aggress. Maltreat. Trauma* 24 674–692. 10.1080/10926771.2015.1049767 29606849 PMC5875428

[B45] Statistics Canada (2018). *Victims of Police-Reported Intimate Partner and Non-Intimate Partner Violence, by Victim Sex, Age Group and Relationship of Accused to Victim, Canada.* Available online at: https://www150.statcan.gc.ca/n1/pub/85-002-x/2019001/article/00018/tbl/tbl02-1-eng.htm (accessed December 30, 2020).

[B46] Straus M. A. (1979). Measuring intrafamily conflict and violence: the Conflict Tactics (CT) Scales. *J. Marriage Fam.* 41 75–86.

[B47] Tabachnick B. G. Fidell L. S. (2013). *Using Multivariate Statistics.* Boston, MA: Pearson.

[B48] Trottier D. Benbouriche M. Bonneville V. (2021). A meta-analysis on the association between rape myth acceptance and sexual coercion perpetration. *J. Sex Res.* 58 375–382. 10.1080/00224499.2019.1704677 31865775

[B49] Wolfe D. A. Scott K. Reitzel-Jaffe D. Wekerle C. Grasley C. Straatman A. (2001). Development and validation of the conflict in adolescent dating relationships inventory. *Psychol. Assess.* 13 277–293. 10.1037/1040-3590.13.2.277 11433803

